# Spontaneous Unilateral Twin Live Ectopic Pregnancy: A Systematic Review of Reported Cases, Diagnostic Challenges, and Management Approaches

**DOI:** 10.7759/cureus.90378

**Published:** 2025-08-18

**Authors:** Meenakshi Sidhar, Neeru Malik, Vijay Dhankar, Dakshika Lochan, Vyomesh Rastogi, Nidhi Prabha Sehgal, Nikhil Talwar, Nikita Madan, Albert A Zomorrodian, Rishabh Kochar

**Affiliations:** 1 Department of Pathology, Dr. Baba Saheb Ambedkar Medical College and Hospital, New Delhi, IND; 2 Department of Obstetrics and Gynecology, Dr. Baba Saheb Ambedkar Medical College and Hospital, New Delhi, IND; 3 Department of Forensic Medicine, Dr. Baba Saheb Ambedkar Medical College and Hospital, New Delhi, IND; 4 Department of Anesthesiology and Critical Care, Dr. Baba Saheb Ambedkar Medical College and Hospital, New Delhi, IND; 5 Department of General Surgery, Lady Hardinge Medical College, New Delhi, IND; 6 Department of Obstetrics and Gynecology, Employees State Insurance Post Graduate Institute of Medical Education and Research, New Delhi, IND; 7 Department of Biological Anthropology, University of California, San Diego, USA; 8 Department of Pulmonary, Sleep, and Critical Care Medicine, All India Institute of Medical Sciences, Jodhpur, IND

**Keywords:** ectopic, extrauterine pregnancy, fetal heart, laparoscopy, pregnancy complications, pregnancy outcome, salpingectomy, tubal pregnancy, twin pregnancy, ultrasonography

## Abstract

Unilateral twin live ectopic pregnancy is a rare and potentially life-threatening obstetric condition, marked by the implantation of two viable embryos within the same fallopian tube. These cases pose significant diagnostic and therapeutic challenges, particularly when fetal cardiac activity is present. This systematic review consolidates existing literature to explore the clinical presentation, underlying pathophysiology, diagnostic modalities, and management strategies associated with this uncommon condition, with a focused discussion on maternal outcomes. A comprehensive literature search was conducted across PubMed, Embase, Scopus, and the Cochrane Database of Systematic Reviews for articles published from January 1945 to May 2025. Both MeSH terms and non-MeSH keywords, including “Unilateral,” "Twin," “Live,” and "ectopic pregnancy," were used, yielding 503 records. After applying inclusion and exclusion criteria and manual screening, 17 case reports published between 1994 and 2023 were included for final analysis. Eligible studies were limited to English-language reports describing spontaneous unilateral twin ectopic pregnancies with confirmed fetal cardiac activity and adequate clinical details. Studies lacking these elements were excluded.

The Joanna Briggs Institute (JBI) Critical Appraisal Checklist for Case Reports was used to assess the risk of bias (ROB) in this study. Due to the descriptive nature of the included studies, formal grading of evidence was not applied. All 17 included cases described women aged 24 to 44 years, typically presenting between six and 12 weeks of gestation with abdominal pain, vaginal bleeding, and amenorrhea. Transvaginal ultrasonography (TVUS) was the most frequently used diagnostic modality, revealing two gestational sacs with cardiac activity within a single fallopian tube. Serum serial β-human chorionic gonadotropin (β-hCG) levels were consistently elevated above the discriminatory threshold. In one particular case, advanced imaging such as magnetic resonance imaging (MRI) was used to clarify anatomy or assess rupture risk. All cases except one were managed surgically, most commonly through laparoscopic salpingectomy or salpingostomy. Methotrexate therapy was attempted in one case and is generally deemed inappropriate due to the presence of viable embryos. No concurrent intrauterine pregnancies were observed. Favorable maternal outcomes were reported in all cases when diagnosis and treatment were timely.

Findings from this review are limited by the rarity of the condition, reliance on isolated case reports, and the lack of standardized diagnostic or treatment protocols. There is also potential for publication bias and underreporting of negative outcomes. Nonetheless, this review emphasizes the importance of early and accurate diagnosis using TVUS, supplemented by MRI when indicated, followed by prompt surgical management to optimize maternal safety. Increased awareness, reporting, and documentation of such rare presentations are crucial to guiding future clinical decision-making and preventing catastrophic complications.

No external funding was received for this review. This review was not registered in any database due to its retrospective, descriptive nature.

## Introduction and background

Ectopic pregnancy is defined as the implantation of a fertilized ovum outside the endometrial cavity, most commonly in the fallopian tube, and remains a principal cause of first-trimester maternal morbidity and mortality [1]. Though ectopic pregnancy occurs in approximately 1%-2% of all pregnancies, twin ectopic pregnancies are extraordinarily rare, with an incidence estimated between one in 125000 and one in 250000 pregnancies [2]. Among these, spontaneous unilateral twin live ectopic pregnancies, where two viable embryos implant within the same fallopian tube, are exceedingly uncommon, with very few well-documented cases showing demonstrable fetal cardiac activity [3,4].

The precise pathogenesis of spontaneous unilateral twin ectopic pregnancies is not fully understood. Proposed mechanisms include two theories: monozygotic twinning post-implantation in the fallopian tube or synchronous implantation of two separate fertilized ova in the same tube. Further, delayed tubal transport of both ova and implantation facilitated by a receptive tubal environment can lead to unilateral twin ectopic conception [5]. Monochorionic, monoamniotic twin pregnancies are always unilateral. However, if the pregnancy is dichorionic and diamniotic, it may still be unilateral, but can rarely present as a bilateral ectopic [6]. The presence of two gestational sacs in the tube significantly increases the risk of tubal rupture due to limited tubal distensibility. Risk factors commonly associated with ectopic pregnancy, such as previous tubal surgery, pelvic inflammatory disease (PID), assisted reproductive technologies (ARTs), and intrauterine device use, are often present; however, some cases arise without identifiable precipitating conditions [4]. Notably, assisted reproductive techniques (ART) such as in vitro fertilization (IVF) have been increasingly associated with an elevated risk of ectopic pregnancy, including rare forms like heterotopic and twin ectopic gestations, due to altered embryo transfer dynamics and tubal pathology.

Clinically, spontaneous unilateral twin live ectopic pregnancy typically presents between six and 12 weeks of gestation, with abdominal pain, vaginal bleeding, and amenorrhea being the most frequent symptoms [1]. Serum β‑HCG levels in twin ectopic gestations commonly surpass the discriminatory threshold for intrauterine detection [3,5]. Transvaginal ultrasonography (TVUS) often reveals two gestational sacs or fetal poles within a single adnexal structure, frequently accompanied by cardiac activity. Doppler ultrasonography, which shows a "ring of fire," may help identify viable ectopic pregnancies [4]. Despite these diagnostic modalities, pre-operative identification remains elusive, and fewer than 10% of cases receive a definitive diagnosis before surgical intervention.

Advanced imaging techniques such as magnetic resonance imaging (MRI) are increasingly being utilized in complex or equivocal cases. T2-weighted and T2-weighted MRI demonstrate high sensitivity and specificity in confirming tubal ectopic pregnancy by identifying signs such as gestational sac-like structures, haemato-salpinx, tubal wall enhancement, and hemoperitonium [7]. 

Surgical management remains the primary treatment in unilateral twin live ectopic pregnancy. Salpingectomy or salpingostomy remains the standard of care when fetal cardiac activity is present, due to the high risk of tubal rupture and hemorrhage, typically via laparoscopy for stable patients or laparotomy for unstable patients [8]. Medical management with methotrexate has been attempted in diagnosed, early, hemodynamically stable cases without fetal cardiac activity. Notably, there are successful reports of both single- and two-dose methotrexate protocols resolving twin tubal ectopic pregnancy [6,9]. A recent case documented spontaneous twin tubal pregnancy resolution following a two-dose methotrexate regimen, with serial β-human chorionic gonadotropin (β-hCG) reduction to <1 mIU/mL by day 31. However, methotrexate remains experimental in this setting, and rupture risk persists [9].

Despite the rarity of spontaneous unilateral twin live ectopic pregnancy, documented maternal outcomes are generally favorable when diagnosis and intervention are prompt. However, delays may lead to tubal rupture, hypovolemic shock, and increased morbidity or mortality [2,6]. Each case contributes valuable clinical insight, given the absence of consensus on optimal medical therapy and long-term reproductive outcomes. This systematic review aims to synthesize existing case reports on unilateral twin live ectopic pregnancy to elucidate incidence, diagnostic methods, management strategies, and maternal outcomes, thereby informing future clinical practice and research directions.

## Review

Materials and methods

This systematic review was conducted in adherence to the Preferred Reporting Items for Systematic Reviews and Meta-Analyses (PRISMA 2020) guidelines, which promote transparency and standardized reporting in systematic reviews [10]. Additionally, the Joanna Briggs Institute (JBI) Critical Appraisal Tools were employed to assess the risk of bias (ROB) and methodological quality of included case reports. These tools are specifically designed to evaluate the rigor of evidence in systematic and integrative reviews [11].

Search Strategy

A comprehensive literature search was conducted across four electronic databases, PubMed, Cochrane Library, Embase, and Scopus, covering the period from January 1, 1945, to June 30, 2025. In PubMed, an advanced search strategy was employed using a combination of MeSH terms (“Pregnancy, Twin” AND “Pregnancy, Ectopic”). The Boolean operator “AND” was applied to ensure that retrieved articles addressed both conditions concurrently. In Embase, Emtree terms such as 'twin pregnancy'/exp AND 'ectopic pregnancy'/exp were used with filters for human subjects and clinical case reports. The Cochrane Library search included MeSH terms and keywords “Twin Pregnancy” AND “Ectopic Pregnancy,” limited to human studies and clinical formats, which yielded no results. For Scopus, a keyword-based search was conducted using TITLE-ABS-KEY("twin pregnancy" AND "ectopic pregnancy") with subject area limited to Medicine (MEDI) and language restricted to English to target relevant content within titles, abstracts, and keywords. A supplementary manual search was also performed using non-indexed terms such as “live birth,” “twin ectopic birth,” and “unilateral twin ectopic pregnancy” to capture rare case reports and grey literature. The search was restricted to English-language studies involving human subjects, with emphasis on clinical publication types such as case reports and case series. A total of 503 records were identified, from which 17 eligible case reports published between 1994 and 2023 were included based on the predefined criteria.

Inclusion and Exclusion Criteria

Studies were selected based on a predefined Population, Intervention, Comparison, Outcome, Study design (PICOS) framework [10]. Inclusion criteria encompassed case reports or case series documenting unilateral live twin ectopic pregnancies, providing sufficient clinical, diagnostic, and therapeutic details, and published in English. Studies were excluded if they involved bilateral or heterotopic pregnancies, non-viable or molar twin ectopic gestations, or if they were reviews, commentaries, or reports lacking adequate clinical information.

Study Selection and Data Extraction

**Figure 1:**
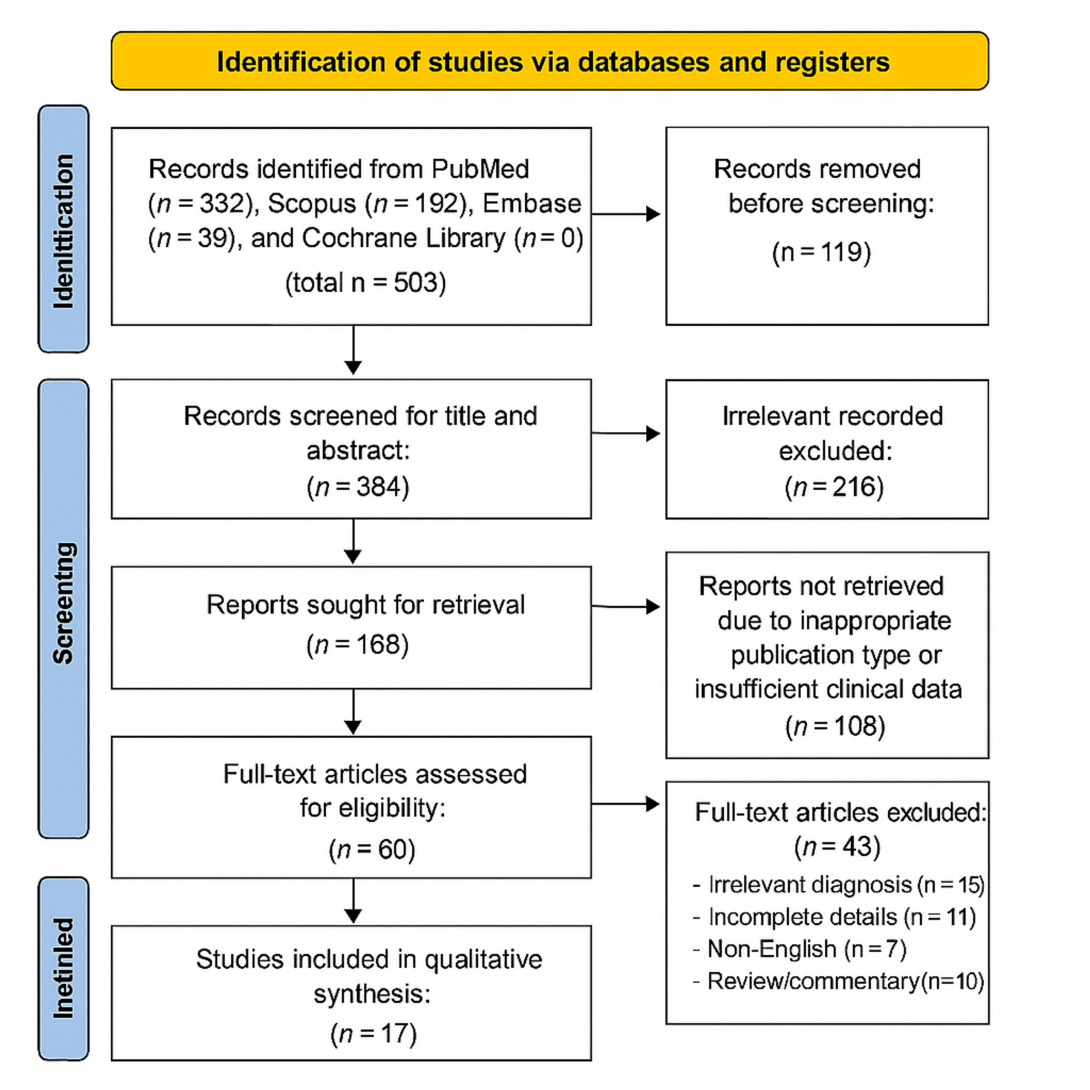
PRISMA 2020 flow diagram for systematic reviews based on searches of databases, registers, and other sources PRISMA: Preferred Reporting Items for Systematic Reviews and Meta-Analyses

A total of 503 records were identified through database and manual searches. After the removal of 119 duplicates, 384 unique records remained. Following title and abstract screening, 216 studies were excluded for being non-English, irrelevant, or not focused on ectopic twin pregnancies, leaving 168 articles for further review. Upon more detailed screening, 108 studies were excluded based on publication type or insufficient clinical detail, and 60 full-text articles were assessed for eligibility. Of these, 43 were excluded for lacking confirmed diagnosis or management data, resulting in 17 eligible full-text articles. Finally, 17 studies published between 1994 and 2023 met all inclusion criteria and were incorporated into the qualitative synthesis. The study selection process is outlined in the PRISMA 2020 flow diagram (Figure 1).

Data were independently extracted by two reviewers using a standardized form that captured key clinical variables, including patient demographics (age, gravidity, and parity), relevant risk factors such as PID, ART use, and history of ectopic pregnancy. Additional details recorded were presenting symptoms, diagnostic imaging findings, surgical observations, treatment modalities employed, and both maternal and fetal outcomes. Any discrepancies encountered during data extraction were resolved through mutual consensus between the reviewers.

Quality Appraisal

ROB was evaluated using the JBI Critical Appraisal Checklist for Case Reports, which assesses elements such as clarity of patient history, diagnostic justification, appropriateness of clinical intervention, and completeness of outcome reporting [12]. Two reviewers independently appraised each study, with discrepancies resolved through discussion. While most case reports demonstrated moderate to high methodological quality, common limitations included a lack of comparator groups, incomplete follow-up data, and potential reporting bias. The detailed appraisal and individual study assessments are summarized in Table 1. 

**Table 1:** ROB assessment (JBI checklist adapted for case reports) ROB: risk of bias; JBI: Joanna Briggs Institute ✓: clearly reported; ✗: not reported; - : incompletely or unclearly reported

Case No.	Author (year)	Demographics reported	History clearly described	Clinical condition at presentation	Diagnostic tests clearly described	Intervention described	Post-intervention outcome stated	Adverse events reported	Takeaway lessons or novel insight	Overall ROB
1	Gualandi M (1994) [13]	✗	✗	✗	✓	✓	✗	✗	✗	High
2	Basama FM (2003) [14]	✓	✓	✓	✓	✓	-	✗	✗	Moderate
3	Sur SD (2005) [15]	✓	✓	✓	✓	✓	✓	✗	✗	Low–Moderate
4	Eddib A (2006) [16]	✓	✓	✓	✓	✓	✓	✗	✗	Low
5	Sergel MJ (2009) [17]	✓	✓	✓	✓	✓	✓	✗	✓	Low
6	Karanjgaokar V (2009) [18]	✓	✓	✓	✓	✓	✓	✓	✓	Low
7	Vohra S (2014) [19]	✓	✓	✓	✓	✓	✓	✗	✗	Low
8	Longoria TC (2014) [20]	✓	✓	✓	✓	✓	✓	✗	✓	Low
9	Ghanbarzadeh N (2015) [21]	✓	✓	✓	✓	✓	✓	✗	✓	Low
10	Kim CI (2018) [22]	✓	✓	✓	✓	✓	✓	✗	✓	Low
11	Seak CJ (2019) [23]	✓	✓	✓	✓	✓	✓	✗	✓	Low
12	Pek JH (2020) [24]	✓	✓	✓	✓	✓	✓	✗	✓	Low
13	Martin A (2021) [25]	✓	✓	✓	✓	✓	✓	✗	✓	Low
14	Jin Li (2021) [26]	✓	✓	-	✓	✓	✓	✗	✓	Low
15	Madaan S (2021) [6]	✓	✓	✓	✓	✓	✓	✗	✓	Low
16	Gure Eticha (2022) [27]	✓	✓	✓	✓	✓	✓	✗	✓	Low
17	Öztürk E (2023) [8]	✓	✓	✓	✓	✓	✓	✗	✓	Low

Results

Demographics and Clinical Presentation

Seventeen published case reports of unilateral live twin ectopic pregnancies conceived spontaneously were identified across diverse countries, including the United Kingdom, the United States, India, Iran, South Korea, and Ethiopia. Patient ages ranged from 24 to 44 years (mean: 30.2 years), with the majority being multigravida. Reported gestational age at diagnosis ranged from six to 12+six weeks. All patients presented with lower abdominal pain; seven reported associated vaginal bleeding. Two patients were hemodynamically unstable due to tubal rupture. No intrauterine gestation was noted in any case, and one patient had no identifiable risk factor, highlighting the diagnostic challenge.

Diagnostic Features and Laterality

TVUS was the primary diagnostic modality in 16 cases, with one case utilizing MRI for further evaluation of chorionicity. Serum β-hCG levels were inconsistently reported but ranged widely (1,750 to 127,040 IU/L), limiting their diagnostic specificity. Chorionicity was confirmed in seven cases: dichorionic diamniotic (DCDA) (n=3), monochorionic diamniotic (MCDA) (n=2), monochorionic monoamniotic (MCMA) (n=2), and not specified in the rest. The right fallopian tube was involved in nine cases, the left in seven, and one case involved a tubal remnant post-salpingectomy. All cases demonstrated two viable gestational sacs with fetal cardiac activity within a single fallopian tube.

Management and Outcomes

Surgical intervention was performed in 16 of 17 cases, most commonly via salpingectomy. Laparoscopy was preferred where feasible, while laparotomy was used in unstable patients or where laparoscopy was contraindicated. Conservative tubal surgery was attempted in three cases. One hemodynamically stable patient was successfully managed medically with methotrexate. Blood transfusion was required in three cases, while one Jehovah’s Witness was managed without transfusion. All patients recovered well, with no maternal mortality or major postoperative complications reported. These findings highlight the importance of early diagnosis and suggest medical therapy may be considered in select stable cases.

A comparative summary of included cases is presented in Table 2.

**Table 2:** Comparative evaluation of reported cases of spontaneous unilateral live twin ectopic pregnancies Laparotomy is an open abdominal surgical approach, laparoscopy is a minimally invasive alternative, and salpingectomy refers to the surgical removal of the fallopian tube. US: ultrasound; MRI: magnetic resonance imaging; TVUS: transvaginal ultrasound; G: gravida; P: para; A: abortion; L: live; DCDA: dichorionic diamniotic; MCDA: monochorionic diamniotic; MCMA: monochorionic monoamniotic; β-hCG: beta human chorionic gonadotropin

Case no.	Author (year)	Country	Patient age	Gravida/para	Gestational age (weeks+days)	Symptoms	Chorionicity	Diagnosis	Tubal side	Management	Outcome
1	Gualandi M (1994) [13]	France	Not specified	Not specified	Not specified	Not specified	Not specified	TVUS	Not specified	Laparotomy	Not specified
2	Basama FM (2003) [14]	UK	31	G2P1L2	7w+2d	lower abdomen pain	Not mentioned	TVUS	Right	Laparscopy+salpingectomy	Only one fetus had cardiac activity; details limited
3	Sur SD (2005) [15]	UK	24	G1	6w	Abdominal pain and brown discharge	Not specified	TVUS+β-hCG (10500 IU/L)	Left	Laproscopy+salpingectomy	No complications
4	Eddib A (2006) [16]	USA	41	G7P4	7w	Abdomen pain	Not specified	TVUS+β-hCG (31672 IU/L)	Right	Laproscopy+salpingectome	No complications
5	Sergel MJ et al. (2009) [17]	USA	24	Not specified	11w+3d	Lower abdomen pain	MCMA	TVUS+β-hCG (60104 IU/L)	Right	Laprotomy+salpingectomy	No complications
6	Karanjgaokar V et al. (2009) [18]	UK	36	G5P2L2A2	7w	Lower abdomen pain+spotting	Not mentioned	TVUS+β-hCG (38900 IU/L)	Left	Laparscopy+ salpingectomy	Jehovah’s Witness; managed without transfusion
7	Vohra S et al. (2014) [19]	UK	34	G3P0A2	6w	Abdominal pain and spotting	not mentioned	TVUS	Left	Laprocopy+salpingectomy	Successful
8	Longoria TC et al. (2014) [20]	USA	44	G5P1L1A2	8w	Pelvic pain	Not specified	TVUS+ β-hCG (21989 IU/L)	Left (tube remnant post-salpingectomy)	Laprocopy+salpingectomy	Twin ectopic in tubal remnant removed without complications
9	Ghanbarzadeh N et al. (2015) [21]	Iran	31	G3P1	Not specified	Abdominal pain and vaginal bleeding	MCMA	TVUS+β-hCG (1750 IU/L)	Right	Laprotomy+salpingectomy	successful management
10	Kim CI et al. (2018) [22]	South Korea	31	G1	7w+7d	Lower abdominal pain, nausea, and vomiting	DCDA	TVUS+β-hCG (35672.3 IU/L)	Right	Laprocopy+salpingectomy	Both fetuses removed successfully; recovery uneventful
11	Seak CJ et al. (2019) [23]	China	37	G2P1	12w+6d	Lower abdominal pain	MCDA	MRI+β-hCG (127040 IU/L)	Right	Laparotomy+salpingectomy	Successful removal of ectopic mass
12	Pek JH et al. (2020) [24]	USA	31	G1	7w	lower abdominal pain	DCDA	TVUS+β-hCG (24271 IU/L)	Right	Laparoscopy+right salpingectomy	No complications
13	Martin A et al. (2021) [25]	Australia	36	G3P1	6w+2d	Pain and vaginal bleeding	not mentioned	TVUS +β-hCG (11 870 IU/L)	Right	Laprocopy+salpingectomy	Resolved successfully; no complications
14	Jin Li et al. (2021) [26]	China	30	G1	8w	Not specified	Not mentioned	TVUS+β-hCG (27036 IU/L)	Right	Laparscopy+salpingectomy	No complications
15	Madaan S et al. (2021) [6]	India	32	G1P1L1A2	7w+2d	Lower abdomen pain	MCDA	TVUS+β-hCG (10000 IU/L)	left	Methotrexate injection in yolk sac	No complications
16	Gure Eticha T (2022) [27]	Ethiopia	30	G3P2	6w+5d	Severe abdominal pain and vaginal bleeding	DCDA	TVUS+β-hCG (86456IU/L)	Left	Laparotomy+salpingectomy	Successful removal of ectopic mass
17	Öztürk E et al. (2023) [8]	Turkey	31	G4P3L3	7w+5d	Lower abdomen pain and vaginal bleeding	Not specified	TVUS+β-hCG (25 696 IU/L)	Left	Laparoscopic+salpingectomy	No complication

Discussion

The occurrence of a spontaneous unilateral live twin ectopic pregnancy represents an extraordinary and perilous deviation from normal implantation, posing significant diagnostic and therapeutic challenges in modern obstetrics. As an exceptionally rare and life-threatening condition, it pushes the boundaries of conventional diagnostic and management protocols. While singleton ectopic pregnancies are already regarded as emergencies due to the risk of rupture and hemorrhage, the presence of two viable fetuses exponentially increases the danger, often resulting in earlier tubal rupture, severe intra-abdominal hemorrhage, and leading to rising maternal morbidity.

The presence of an adnexal mass, vaginal bleeding, and nonspecific lower abdominal pain constitutes the classic symptom triad of ectopic pregnancy. However, only 45% of ectopic pregnancy cases exhibit all three symptoms [3]. In all reviewed cases, high-resolution TVUS was pivotal in early diagnosis, successfully identifying two gestational sacs with distinct fetal cardiac activity within a single fallopian tube, with a reported sensitivity of 87.0%-99.0% and specificity of 94.0%-99.9% [5]. Here, the role of color Doppler ultrasonography becomes increasingly vital. Doppler interrogation often reveals a “Ring of Fire” appearance, characterized by hypervascular peri-trophoblastic flow surrounding the gestational sac, which aids in distinguishing unruptured ectopic pregnancies from corpus luteum cysts and other adnexal masses [4,28]. This sign is especially critical in viable ectopic pregnancies where timely intervention can be life-saving.

Serum β-hCG levels may significantly exceed the established discriminatory threshold of 1,500-2,000 mIU/mL for singleton ectopic pregnancies, due to the increased amount of trophoblastic tissue [5]. Notably, the levels may resemble those seen in typical intrauterine pregnancies. In the absence of an intrauterine gestational sac and with typically rising β-hCG levels, the possibility of twin ectopic implantation, though rare, must be considered.

The role of MRI was reported in one case, indicating its limited utility due to cost, accessibility, and time constraints, especially in acute settings [7]. While standardized management protocols for singleton ectopic pregnancy are available from leading institutions such as the American College of Obstetricians and Gynecologists (ACOG), the Royal College of Obstetricians and Gynaecologists (RCOG), and the National Institute for Health and Care Excellence (NICE), none specifically address twin ectopic pregnancies, primarily due to their rarity and lack of inclusion in large datasets [29-31].

We conducted a literature review of unilateral live multiple ectopic pregnancies to compare management decisions and outcomes. We identified 17 cases of spontaneous, live, twin ectopic pregnancies that highlighted key epidemiological, diagnostic, and management-related features of this rare clinical entity. The majority of patients fell within the reproductive age group of 30-37 years, with the youngest being 24 and the oldest 44, an age range that typically corresponds to increased parity and cumulative exposure to risk factors associated with ectopic pregnancies. A range of gravidity was noted, from primigravida to as high as G7P4, suggesting that both first-time pregnancies and high-parity women are affected [16].

Gestational age at diagnosis ranged from six weeks to nearly 13 weeks, with most diagnoses occurring between six and eight weeks of gestation. This aligns with the period when tubal ectopic pregnancies typically become symptomatic due to the limited distensibility of the fallopian tube [1]. However, delayed presentations were also documented, including one case identified at 12 weeks and six days of gestation, highlighting the diagnostic challenges in atypical cases [23]. The most commonly reported presenting symptoms included lower abdominal pain and vaginal bleeding or spotting. These were occasionally accompanied by nausea and vomiting. These nonspecific symptoms emphasize the importance of a high index of suspicion, particularly in early pregnancy with elevated β-hCG levels and inconclusive intrauterine findings on ultrasound.

TVUS combined with serum β-hCG measurement served as the cornerstone of diagnosis in most cases. Serum β-hCG levels were notably high across the series, ranging from 1750 IU/L to 127040 IU/L, frequently exceeding levels typically observed in singleton ectopic pregnancies. In complex or late-presenting cases, MRI was employed to provide additional anatomical detail, underscoring its role in selected cases where TVUS is inconclusive.

Where documented, chorionicity varied across cases. DCDA and MCDA pregnancies were the most frequently identified types. The presence of both DCDA and MCDA configurations suggests that twin tubal pregnancies may result from both dizygotic and monozygotic twinning mechanisms [5]. However, chorionicity was not reported in several cases, pointing to a potential area for improved documentation in future reports. Further analysis revealed a predominance of right-sided tubal involvement, with nine out of 17 cases affecting the right tube. This may reflect the physiological predominance of ovulation from the right ovary or anatomical differences in tubal architecture and vascularization [32]. A study of Xia et al. [33] stated that pooling of blood in the left ovary, possibly due to anatomical characteristics, could cause a temperature increase in the left ovary, subsequently decreasing the potential fertility of oocytes on the left side. Thus, they emphasized that ectopic pregnancy is more frequently located in the right fallopian tube, rather than the left tube. 

Several cases featured noteworthy characteristics. A rare case of twin ectopic pregnancy occurring in a tubal remnant post-salpingectomy was documented, highlighting the importance of considering ectopic implantation even in previously operated tubes [20]. One patient belonging to the Jehovah’s Witness faith was successfully managed without blood transfusion, emphasizing the need for individualized care based on patient values [18]. 

The incidence of tubal rupture is reported to be approximately 32%, with the risk of rupture increasing by roughly 2.5% for each 24 hours when left untreated [34]. To date, the surgical approach remains the most documented method in the literature for treating unilateral tubal twin pregnancies [25]. In our analysis, surgical intervention was the principal mode of management. Laparoscopic salpingectomy was the preferred approach in the majority of cases due to its minimally invasive nature and favorable outcomes [35]. Laparotomy was reserved for selected cases, particularly those with hemodynamic instability, advanced gestational age, or larger ectopic masses. Only one case, reported by Madaan et al., utilized conservative management with direct methotrexate injection into the yolk sac under ultrasound guidance, signifying that non-surgical approaches may be feasible in highly selected, stable patients with early gestation and careful monitoring [6]. Betti et al. [5] and Berkes et al. [36] reported cases of non-viable twin ectopic pregnancies that were treated using a multi-dose intramuscular methotrexate regimen. In both instances, treatment led to tubal rupture and significant intra-abdominal bleeding, ultimately necessitating surgical intervention. The initial serum β-hCG levels were notably high, exceeding 5000 IU/L, measured at 13217 IU/L [36] and 60348 IU/L [5]. These outcomes highlight the need for cautious use of intramuscular methotrexate in twin ectopic gestations, weighing the goal of fertility preservation against the elevated risk of rupture and potential treatment failure.

In summary, this analysis reveals that unilateral twin tubal ectopic pregnancies, though rare, exhibit recognizable clinical patterns. Early gestational age, high β-hCG levels, right-sided predominance, and the frequent need for surgical intervention are consistent findings. With timely diagnosis and individualized management, outcomes are uniformly favorable. Improved documentation of chorionicity, imaging modalities, and follow-up outcomes in future reports may further enhance our understanding of this rare but clinically significant obstetric condition. 

Based on these outcomes and supporting literature, unilateral twin live tubal ectopic pregnancies are rare. While a systematic review of randomized trials found that fertility rates are similar after treatment of ectopic pregnancies with either salpingostomy, salpingectomy, or intramuscular methotrexate, women with a single fallopian tube or bilateral tubal ectopic pregnancies may benefit from fertility preservation with salpingostomy or methotrexate therapy [25]. However, the outcome after ectopic pregnancy is proven to be better after salpingectomy [37]. In the absence of specific guidelines or recommendations, surgical intervention appears to be the most appropriate course of management. Laparoscopy is preferred over laparotomy in hemodynamically stable patients, reflecting modern minimally invasive trends. Intramuscular/intra-gestational methotrexate injection should be used with caution in women with multigestational ectopic pregnancies, as it carries a higher risk of rupture and potential treatment failure [5,25]. While it may help preserve fertility, patients must receive comprehensive counseling about these associated risks before proceeding with medical management.

## Conclusions

Spontaneous unilateral live twin unruptured ectopic pregnancy is an exceptionally rare but life-threatening condition that warrants prompt recognition and intervention. Clinicians must maintain a high index of suspicion in early pregnancy, especially in patients with disproportionately elevated β-hCG levels. High-resolution transvaginal Doppler ultrasonography is the most effective diagnostic tool, capable of detecting dual gestational sacs with cardiac activity within a single fallopian tube. Medical management with methotrexate is relatively contraindicated in live ectopic pregnancies, and even more so in twin live ectopic pregnancies, due to the increased risk of rupture and hemorrhage. Surgical intervention, most commonly salpingectomy, is the universally accepted approach, with laparoscopy preferred in hemodynamically stable patients. Favorable maternal outcomes with minimal morbidity have been consistently reported.

Each case adds to a limited body of evidence that can inform clinical practice and guide management strategies. Despite the absence of formal clinical guidelines for management, this review emphasizes the urgent need for their inclusion in future updates. With the rising use of ARTs, the incidence of multifetal ectopic pregnancies may increase, necessitating greater clinical awareness, early diagnosis, and timely surgical management to ensure optimal outcomes.

## References

[1] Flanagan HC, Duncan WC, Lin CJ, Spears N, Horne AW (2023). Recent advances in the understanding of tubal ectopic pregnancy. Fac Rev.

[2] Sai S, Segu B, Niranjana B (2025). Acute abdomen: an obstetric nightmare in surgical ward. Int J Sci Res.

[3] Atef GM, Al-Attas MA, Alnaqla HS, Alshammari IF (2024). Unilateral tubal twin ectopic pregnancy: a rare case report and literature review. Oman Med J.

[4] Fernandez H, Bourget P, Lelaidier C, Frydman R, Job-Spira N (1993). Methotrexate treatment of unilateral twin ectopic pregnancy: case report and pharmacokinetic considerations. Ultrasound Obstet Gynecol.

[5] Betti M, Vergani P, Damiani GR (2011). Unilateral tubal twin ectopic pregnancy treated with single-dose methotrexate. Arch Gynecol Obstet.

[6] Madaan S, Jaiswal A, Banode P, Dhok A, Dewani D (2021). Spontaneous twin ectopic pregnancy managed successfully with methotrexate-mediated ultrasound-guided fetal reduction: a fertility-preserving approach. Cureus.

[7] Srisajjakul S, Prapaisilp P, Bangchokdee S (2017). Magnetic resonance imaging in tubal and non-tubal ectopic pregnancy. Eur J Radiol.

[8] Öztürk E, Aktürk H (2023). Sonographically positive fetal heartbeat in unilateral tubal twin pregnancy as a rare case with literature review. Cureus.

[9] Forbes LA, Nuthivana N, Morales R (2024). Sonographically positive fetal heartbeat in unilateral tubal twin pregnancy as a rare case with literature review. Case Rep Obstet Gynecol.

[10] Page MJ, McKenzie JE, Bossuyt PM (2021). The PRISMA 2020 statement: an updated guideline for reporting systematic reviews. Br Med J.

[11] Moola S, Munn Z, Tufanaru C (2024). JBI Manual for Evidence Synthesis. JBI Manual for Evidence Synthesis.

[12] Ouzzani M, Hammady H, Fedorowicz Z, Elmagarmid A (2016). Rayyan-a web and mobile app for systematic reviews. Syst Rev.

[13] Gualandi M, Steemers N, de Keyser JL (1994). First reported case of preoperative ultrasonic diagnosis and laparoscopic treatment of unilateral, twin tubal pregnancy (French). Revue Francaise de Gynecologie et D'obstetrique.

[14] Basama FM (2003). Preoperative diagnosis of unilateral tubal twin ectopic pregnancy with one live twin. J Obstet Gynaecol.

[15] Sur SD, Reddy K (2005). Spontaneous unilateral tubal twin pregnancy. J R Soc Med.

[16] Eddib A, Olawaiye A, Withiam-Leitch M, Rodgers B, Yeh J (2006). Live twin tubal ectopic pregnancy. Int J Gynaecol Obstet.

[17] Sergel MJ, Greenberg DT (2009). Live twin ectopic pregnancy with advanced gestation. J Emerg Med.

[18] Karanjgaokar V, Shah P, Nicholson Y, Spence-Jones C (2009). Laparoscopic management of a ruptured unilateral live twin ectopic pregnancy in a Jehovah's Witness. J Obstet Gynaecol.

[19] Vohra S, Mahsood S, Shelton H, Zaedi K, Economides DL (2014). Spontaneous live unilateral twin ectopic pregnancy - A case presentation. Ultrasound.

[20] Longoria TC, Stephenson ML, Speir VJ (2014). Live unilateral twin ectopic pregnancy in a fallopian tube remnant after previous ipsilateral salpingectomy. J Clin Ultrasound.

[21] Ghanbarzadeh N, Nadjafi-Semnani M, Nadjafi-Semnani A, Nadjfai-Semnani F, Shahabinejad S (2015). Unilateral twin tubal ectopic pregnancy in a patient following tubal surgery. J Res Med Sci.

[22] Kim CI, Lee TY, Park ST, Kim HB, Park SH (2018). A rare case of spontaneous live unilateral twin tubal pregnancy with both fetuses presenting with heart activities and a literature review. Obstet Gynecol Sci.

[23] Seak CJ, Goh ZN, Wong AC, Seak JC, Seak CK (2019). Unilateral live twin tubal ectopic pregnancy presenting at 12 weeks of gestation: a case report. Medicine (Baltimore).

[24] Pek JH, Simpson WL Jr, Owen J, Nelson B (2020). Live twin ectopic pregnancy. J Emerg Med.

[25] Martin A, Balachandar K, Bland P (2021). Management of a spontaneously conceived live unilateral twin ectopic pregnancy in Australia: a case report. Case Rep Womens Health.

[26] Li J, Sun W, Jin X, Fei X (2021). A live tubal twin pregnancy. Arch Gynecol Obstet.

[27] Gure Eticha T (2022). Unilateral twin ectopic pregnancy: a case report from the eastern part of Ethiopia, Harar. Int Med Case Rep J.

[28] Summa B, Meinhold-Heerlein I, Bauerschlag DO, Jonat W, Mettler L, Schollmeyer T (2009). Early detection of a twin tubal pregnancy by Doppler sonography allows fertility-conserving laparoscopic surgery. Arch Gynecol Obstet.

[29] American College of Obstetricians and Gynecologists (2018). ACOG practice Bulletin No. 193: tubal ectopic pregnancy. Obstet Gynecol.

[30] Royal College of Obstetricians and Gynaecologists (2016). Diagnosis and management of ectopic pregnancy: green-top guideline No. 21. BJOG.

[31] (2019). National Institute for Health and Care Excellence (NICE). Ectopic pregnancy and miscarriage: diagnosis and initial management. https://www.nice.org.uk/guidance/ng126.

[32] Şahin B, Tinelli A (2022). Tubal ectopic pregnancy in acute abdominal presentation: a case control analysis. Ulus Travma Acil Cerrahi Derg.

[33] Xia W, Zhang J, Zhang D (2019). Left-right asymmetry of tubal pregnancy: a 12-year retrospective hospital-based study. J Minim Invasive Gynecol.

[34] Bickell NA, Bodian C, Anderson RM, Kase N (2004). Time and the risk of ruptured tubal pregnancy. Obstet Gynecol.

[35] Singh S, Sandhu N, Singh S (2020). Comparison between laparoscopy and laparotomy in the management of ectopic pregnancy: a retrospective study. Int J Reprod Contracept Obstet Gynecol.

[36] Berkes E, Szendei G, Csabay L, Sipos Z, Joo JG, Rigo J Jr (2008). Unilateral triplet ectopic pregnancy after in vitro fertilization and embryo transfer. Fertil Steril.

[37] Ozcan MC, Wilson JR, Frishman GN (2021). A systematic review and meta-analysis of surgical treatment of ectopic pregnancy with salpingectomy versus salpingostomy. J Minim Invasive Gynecol.

